# Automatic stage identification of *Drosophila* egg chamber based on DAPI images

**DOI:** 10.1038/srep18850

**Published:** 2016-01-06

**Authors:** Dongyu Jia, Qiuping Xu, Qian Xie, Washington Mio, Wu-Min Deng

**Affiliations:** 1 Department of Biological Science, Florida State University, Tallahassee, FL 32306-4370, USA; 2 Department of Mathematics, Florida State University, Tallahassee, FL 32306-4510, USA; 3 Department of Statistics, Florida State University, Tallahassee, FL 32306-4330, USA

## Abstract

The *Drosophila* egg chamber, whose development is divided into 14 stages, is a well-established model for developmental biology. However, visual stage determination can be a tedious, subjective and time-consuming task prone to errors. Our study presents an objective, reliable and repeatable automated method for quantifying cell features and classifying egg chamber stages based on DAPI images. The proposed approach is composed of two steps: 1) a feature extraction step and 2) a statistical modeling step. The egg chamber features used are egg chamber size, oocyte size, egg chamber ratio and distribution of follicle cells. Methods for determining the on-site of the polytene stage and centripetal migration are also discussed. The statistical model uses linear and ordinal regression to explore the stage-feature relationships and classify egg chamber stages. Combined with machine learning, our method has great potential to enable discovery of hidden developmental mechanisms.

Female *Drosophila* have two ovaries, each containing around 16 ovarioles. The ovariole is a string of 6 or 7 sequentially developing egg chambers. Each *Drosophila* egg chamber is a basic developmental unit of oogenesis. Furthermore, the egg chamber comprises 16 germ-line cells, one oocyte and 15 nurse cells, which are surrounded by a thin layer of somatic follicle cells^1^. Research on the egg chamber contributes significantly to scientific knowledge. The egg chamber follicle cell epithelium has been a well-established model system to study cell cycle regulation, cell differentiation^2^,^3^,^4^,^5^,^6^,^7^,^8^,^9^,^10^,^11^,^12^,^13^,^14^,^15^,^16^, cell polarity^17^,^18^,^19^,^20^, endocytosis^21^,^22^,^23^, exocytosis^24^, morphogenesis^19^,^25^,^26^,^27^,^28^, and cancer metastasis^29^,^30^. Germline-soma interactions provide developmental cues, especially for anterior-posterior and dorsal-ventral patterning^31^,^32^,^33^,^34^,^35^,^36^. More interestingly, egg chambers have distinctively separated germline and soma, which provides a unique and reliable system to study germline-soma ligand-receptor interaction^1^,^2^,^31^,^37^.

The development of the egg chamber during *Drosophila* oogenesis is divided into 14 stages^1^. Visual identification of different stages requires significant training, including a thorough understanding of egg chamber growth progression. Even skilled scientists need to cautiously determine the stages based on general features (Fig. 1)^1^ and frequently refer to neighboring egg chambers to doubly confirm the determination of stages. However, in a single confocal image of egg chambers, there might be no neighboring egg chambers to help confirm stages. Moreover, even an expert sometimes has difficulty assigning correct stages to inter-stage transitional egg chambers. To address these problems, and to reduce the bias caused by human perceptual variation, an objective, reliable and standardized egg chamber stage identification method is greatly needed.

DAPI (4′,6-diamidino-2-phenylindole) is widely used as a cell nucleus staining dye. In this study, we developed a toolbox that applies understanding of egg chamber morphology^1^ extracted automatically from DAPI staining to determine egg chamber stages. We collected 172 confocal microscopy images from different stages to train and evaluate our automatic stage identification method. Egg chamber areas of DAPI images proved to be a stable feature for stage differentiation, while oocyte size played a critical role for late stage differentiation. Integrated with machine learning, our approach provides potential to discover hidden developmental mechanisms, and brings the *Drosophila* community tools for standardizing stage identification and objectively comparing results obtained from different labs. In this paper, we applied our method to successfully confirm the occasional appearance of Broad expression as early as stage 5, and also unambiguously demonstrated that egg chambers with germline *Delta* mutation entered midoogenesis, even though the follicle cells did not show the appropriate biomarker, Br 5. Furthermore, we provided support for the previous understanding that follicle cell mitosis ceases at stage 6^1^,^38^. Our results clearly showed mitosis occurs in follicle cells of stage-5 egg chambers, while not in those of stage-6 egg chambers. Our findings clarified our understanding that stage 6 should be considered as an entering stage of endocycle, and stage 7 completes endocycle entry^2^,^3^,^4^,^5^,^6^,^9^,^10^.

## Results

### Egg chamber morphological feature extraction

**Figure 1:**
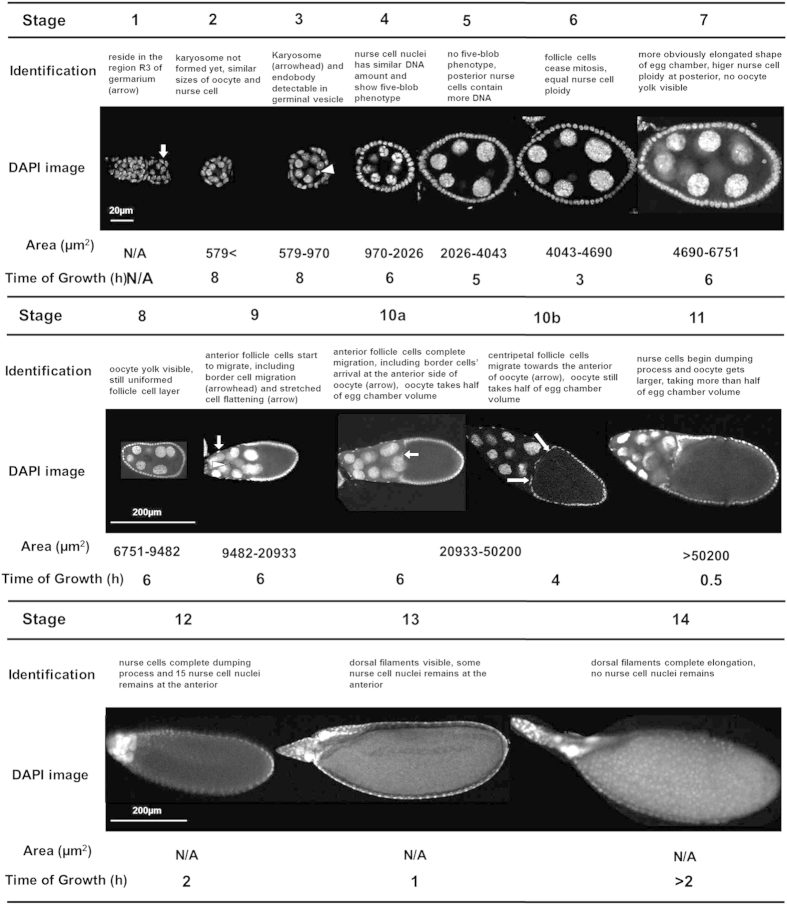
Identification of 14 stages of *Drosophila* egg chamber. Characteristics of different egg chamber stages.

The collected DAPI images should be single cross sections in the middle plane, and represents the largest area size. Before image processing, particular stages were initially assigned to egg chambers by skilled biologists in our team based on standard descriptive guidance^1^ (Fig. 1). The morphological features, including egg chamber size, ratio, orientation, oocyte size, follicle cell distribution, blob-like chromosomes in polytene nuclei at stage 4, centripetal cell migration at stage 10B, were then extracted to train our machine learning method.

### Egg chamber size

**Figure 2:**
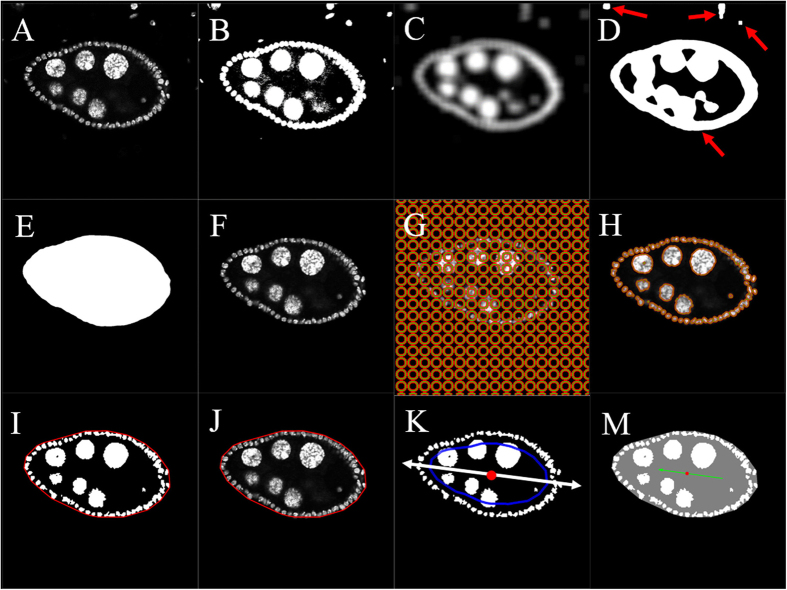
Extraction procedure for egg chamber size, egg chamber ratio and egg chamber orientation. (**A**) Original image. (**B**) First post-thresholding image using Otsu’s method. (**C**) Image after average filter. (**D**) Second post-thresholding image using Otsu’s method on (**C**). There were four connected regions in this image (red arrows). The largest one occupied the area of the egg chamber. The rest were considered noise in the following steps and the noise needed to be removed. (**B–D**) Pre-processing of image to remove noise and identify target area. (**E**) Image mask for the target egg chamber region. (**F**) Identified target egg chamber region in original image intensity. After this step, most noise (smaller region in Fig. 2D) outside the egg chamber was removed. (**G,H**) Initial and final outlines detected from Chan-Vese algorithm (**I**) Detected image mask for egg chamber nuclei and boundary of convex hull of the nuclei. Boundary of egg chamber was indicated in red. (**J**) The final denoised image. This shows the detected egg chamber region in original intensity with outer boundary. User has the flexibility of tuning these parameters based on visual inspection of the final denoised image. (**K**) Shrunken boundary and the major axis of PCA. The outer boundary was shrunk inward to avoid the follicle cells (shrunken boundary was indicated as blue curve). The major axis (white arrow) along posterior-anterior direction was computed based on pixels within the shrunken region. The centroid of the egg chamber was shown as the red dot. (**M**) Detected egg chamber orientation. The green arrow points to the anterior side, which contained more nurse cells.

The development of an organism is often accompanied by increase in size, and this is also the case in *Drosophila* egg chamber stage development. Since egg chamber size plays a critical role in stage determination, we developed an algorithm for automated egg chamber size measurement, whose workflow is described as follows:

Image thresholding: Original images (Fig. 2A) were read into Matlab, and the measurement (μm) of each pixel was recorded. Otsu’s method^39^ was used to convert the original image into a binary image and initialize the segmentation process. Briefly, Otsu’s method applies a threshold that optimizes the separation of image foreground and background. This algorithm has been widely used in image segmentation pre-processing steps^40^. Conditions must be met for Otsu’s method to be effective:1) minimum variability in gray levels of foreground pixels and background pixels, respectively; 2) maximum gray level contrast between background and foreground pixels. In our study, the DAPI-stained images satisfy both conditions, as foreground pixels had much lighter color than the background pixels. We set the threshold at 20% of Otsu’s threshold to include all desired foreground objects. This percentage value was learned through experimentation with data. The Matlab function, graythresh, was used to compute Otsu’s threshold. Another Matlab function, im2bw, applied our threshold and converted the grayscale image to a binary image. The output binary image replaced all pixel values in the input image with luminance greater than the threshold with the value 1 (white, foreground, Fig. 2B) and the remaining pixels with the value 0 (black, background, Fig. 2B).

Largest component extraction: Digital images are prone to noise that may originate from different parts of the image acquisition process. “Noise” is always a relative term and means “unwanted signals”. In our analysis of the binary image obtained in the previous step, we considered any foreground image element other than the egg chamber of interest as noise, including portions of other egg chambers and scattered spotty pixels (Fig. 2A). To detect these noisy pixels and mitigate their negative effects on our analyses, we applied an adjusted average filter, which smoothed the image by replacing each pixel value with the average of the pixel values in its neighborhood. The average filter had the effect of changing the binary (Fig. 2B) into a new grayscale image (Fig. 2C), which connected previously disconnected regions of the “true foreground”, whereas the small noisy areas of the foreground remained disconnected (Fig. 2C). Otsu’s method was re-applied to convert the image (Fig. 2C) back into binary format (Fig. 2D). The Matlab function, regionprops, was then used to identify the pixels in each connected region of the foreground by sequentially labeling the pixels in each such region by their unique indexes once the foreground pixels were identified, and we only kept those in the largest region in the foreground because the egg chamber occupies the largest area. This removed unwanted noisy, scattered pixels. Next, we filled in this largest region, as indicated in Fig. 2E, and used it as a mask on the original image to obtain a denoised image of the egg chamber (Fig. 2F). In other words, for each foreground pixel of the mask (Fig. 2E), the intensity was set to the value in the original image and all other pixels were set to zero (black).

Chan-Vese segmentation method: The Chan-Vese segmentation algorithm^41^ has been successfully used in a wide range of medical applications in lesion and tumor segmentation^42^,^43^,^44^.The Chan-Vese algorithm segments an image by minimizing an energy function that balances out foreground-background boundary length, foreground area, and purity of the foreground and background. We applied the algorithm to segmentation of the gray scale image from the previous step (Fig. 2F). In this algorithm, the initialization of the nuclei boundary can be quite arbitrary, so we selected a collection of small disjointed circles (Fig. 2G), since a set of small loops comprises the nuclei boundary. Figure 2H shows the detected nuclei boundary. Since the egg chamber forms a nearly convex structure, the Matlab function, convhull, was applied to identify all the pixels belonging to the convex hull of the nuclei. The convex hull was identified as the region inside the red curve in Fig. 2I. Thus, the area of the egg chamber was estimated as the area of the convex hull, computed by multiplying the number of pixels in the segmented egg chamber by the area of a single pixel. As in Step 2, we used the binary image shown in Fig. 2I to mask the original image and the result is shown in Fig. 2J. This final denoised image (Fig. 2J) may be used to assess the quality of the denoising process. The default parameter values in the algorithm were chosen based on the superior results that they produced on a large set of DAPI images that guided the development of the method. However, the flexibility of the technique allows the use to tune these parameters based on visual inspection of the final denoised image (Fig. 2J).

### Egg chamber ratio

In the previous section, the shape of the egg chamber was modeled as the convex hull of the cell nuclei, that is, the collection of all pixels within the red curve in Fig. 2I. Thus, the estimation of egg chamber ratio was based on properties of the convex hull. Principal component analysis (PCA)^45^ was applied to the coordinates of the pixels within the convex hull to identify the directions of the axes of major and minor variation. The egg chamber ratio was measured as the ratio of the standard deviations of the data projected onto the major and minor axes, respectively. Since the standard deviation (SD) is a measure that is used to quantify the dispersion of a set of data values, the ratio of SDs measures the ratio of the widths spreading along the major and minor axes. We used this measurement instead of using the lengths along these two directions because standard deviations are much more robust to localized changes in egg chamber shape than absolute lengths. This stability property was extremely important in dealing with tilted images.

### Egg chamber orientation

The major axis of the egg chamber calculated with PCA in the section of “egg chamber ratio” aligned well with the oriented posterior-anterior axis. The middle axis along the major axis was constructed to determine the orientation of egg chamber. However, PCA just gives an axis, not an orientation. To decide the orientation, we radially shrank the boundary of the egg chamber inwards about the centroid (red dot in the middle of egg chamber in Fig. 2K) of the chamber (blue curve in Fig. 2K) to keep the follicle cells on the exterior. The anterior end was characterized as the side of the chamber that contained a larger number of nurse cells. Figure 2M indicates the orientation obtained with this method. This equipped the egg-chamber image with a posterior-anterior directionality (green arrow in Fig. 2M).

### Oocyte size

**Figure 3:**
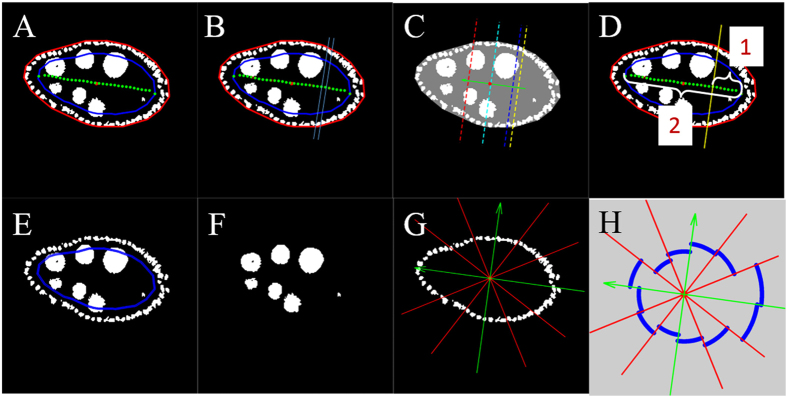
Determination of oocyte size and follicle cell distribution. (**A**) Constructed middle axis (green curve) in binary image along posterior-anterior direction with each point numerically ordered along P-A direction. (**B**) One example of band (in cyan). The boundaries of this band were perpendicular to the major axis and went through two consecutive points on the middle axis. (**C**) Detected oocyte region boundary (yellow line). The oocyte boundary was a line perpendicular to the major axis and passed the point closest to the posterior end such that the fraction of foreground pixels in the discrete band was at least 10%. (**D**) Illustration of the length proportion of the oocyte region. This length proportion was defined as the quotient of middle axis length within the oocyte region (1) and the whole middle axis length (2). (**E**) Adjustable shrunken boundary (blue curve) which passed follicle cells. (**F**) Detected mask of nurse cells. (**G**) Detected follicle cells were divided into 12 equal angle sectors (green arrows show posterior-anterior direction and dorsal-ventral direction). (**H**) Visualization of follicle cell distribution. Circle-like blue curves indicate more uniform distribution of follicle cells.

The oocyte localizes to the posterior side of the egg chamber and we approximated its boundary by a line segment perpendicular to the posterior-anterior axis. To estimate the boundary, we sampled the middle axis along the posterior-anterior axis at 30 equally spaced points *Q*_*1*_, ···, *Q*_*30*_, starting from the posterior end (Fig. 3A). For each *i,* we constructed a band perpendicular to the posterior-anterior axis bounded by segments through *Q*_*i*_ and *Q*_*i+1*_ (one such band is shown in Fig. 3B) and counted the fraction of foreground pixels within that band. We set the oocyte boundary (yellow line in Fig. 3C) to be determined by the segment perpendicular to the axis through the point *Q*_*i*_ closest to the posterior end such that the fraction of foreground pixels was at least 10%. The portion of egg chamber area that corresponds to the oocyte was computed as the ratio of the number of pixels belonging to the oocyte region and the number of pixels belonging to the whole egg chamber. For visualization purposes, the blue, green and red lines in Fig. 3C show the segments perpendicular to the posterior-anterior axis for which 1/3, 1/2 and 2/3, respectively, of the whole egg chamber area lies on the posterior side. Although not used in this study, another measurement that can be derived from this calculation is the proportion of the posterior-anterior axis that falls in the oocyte region (Fig. 3D).

### Follicle cell distribution

To estimate the distribution of follicle cells we needed to discern them from the nurse cells. To initialize the process, as in the section of “egg chamber orientation”, we shrunk the boundary of the egg chamber inward (blue curve in Fig. 3E) by an amount determined by the egg-chamber size. Since the nurse cells are much larger than follicle cells, only follicle cells fall completely outside the shrunken boundary, even though some nurse cells might intersect the boundary, thus allowing separation of the follicle cells (Fig. 3G). The distribution of the follicle cells was quantified as follows. We set a coordinate system based at the centroid of the egg chamber using the posterior-anterior axis and an axis perpendicular to it. We then divided the plane into 12 sectors of equal width. Within each sector we calculated the average follicle cell density, defined as the number of foreground pixels within that sector divided by the length of the part of the egg chamber boundary within the sector. The distribution of average follicle cell density was depicted as indicated in Fig. 3H. This representation gave us a visual tool to inspect the uniformity of the distribution of follicle cells. Circle-like curves (blue in Fig. 3H) indicate a more uniform distribution of follicle cells. To quantify the uniformity of the distribution, we used the ∆-distance^46^ between the learned distribution and the uniform distribution defined as 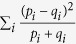, where 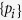,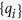,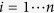 where *p* and *q* are vectors representing two discrete distributions. Follicle cells distribution of egg chambers in different stages is shown in Supplementary Figs 1, 2 and 3.

### Blob-like chromosomes in polytene nuclei at stage 4

**Figure 4:**
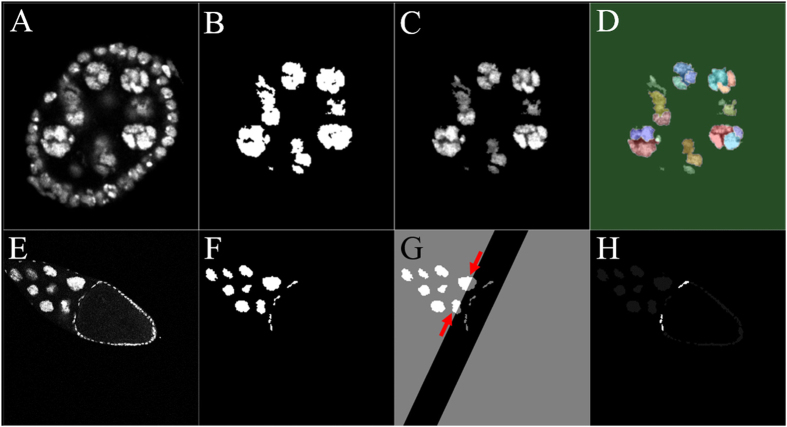
Detection of blob-like chromosomes in polytene nuclei and centripetal cell migration. (**A**) Original image of a stage-4 egg chamber. (**B**) Detected mask of nurse cells. (**C**) Detected nurse cells in original intensities. (**D**) Watershed algorithm output. Each nurse cell had been further divided into several small parts denoted by different colors. This varied coloration indicates the existence of blob-like chromosomes in polytene nuclei. (**E**) Original image of a stage-10B egg chamber. (**F**) Detected mask for inside cells. (**G**) Possible centripetal migration region was highlighted as adjustable black band perpendicular to the major axis. In this picture, part of two nurse cells were captured in this band (red arrow), and since they only partially belonged to this band, the algorithm excluded those as candidates of centripetal migration cells. (**H**) Detected centripetal cell migration. Centripetal cell migration was identified as whole cells inside the band near the outer boundary.

Here we describe a method that was used to detect blob-like chromosomes in polytene nuclei for stage-4 egg chambers. For a given image, as shown in Fig. 4A, following the previous steps we constructed a binary mask for the nurse cell nuclei (Fig. 4B) that was applied to the original image to segment the nuclei of the nurse cells (Fig. 4C). In this way, we extracted the nurse-cell nuclei with the original image intensities (Fig. 4C). Then this image was read into a marker-controlled watershed segmentation algorithm^47^. This algorithm has been used in many applications that require separation of touching objects^48^,^49^. The watershed transformation found “catchment basins” and “watershed ridge lines” in the image by treating it as a topographical relief, where the gray-level of a pixel was treated as its height in the relief. From this watershed segmentation result (Fig. 4D), we could see that almost every nurse cell had been further divided into several small parts indicated by different colors in Fig. 4D.

### Centripetal cell migration at stage 10B

To differentiate egg-chamber stages 10A and 10B, we used an algorithm that detected the centripetal follicle cells. For a given image (Fig. 4E), the previous steps for constructing a mask for the nurse cells were applied. The resulting binary image also includes the nuclei of the centripetal follicle cells (Fig. 4F). To remove the nurse cells from the binary mask, we applied the same technique used for oocyte size estimation and employed the result from PCA in the section of “egg chamber ratio”. We first identified a band with adjustable width by detecting the oocyte boundary (Fig. 4G). We set the bandwidth at 0.8 of the standard deviation along the major axis, so as to capture all possible migrating cells without including an entire nurse cell in the band. We then detected the cells that were close to the egg chamber boundary as the centripetal cells (highlighted in Fig. 4H).

### Statistical analysis between morphology and stage

In the statistical analysis section, for modeling purposes, we combined specimens of stage 10A and stage 10B as stage 10. Further separation of these two stages can be done using the centripetal migration feature.

### Boxplot and regression

**Figure 5:**
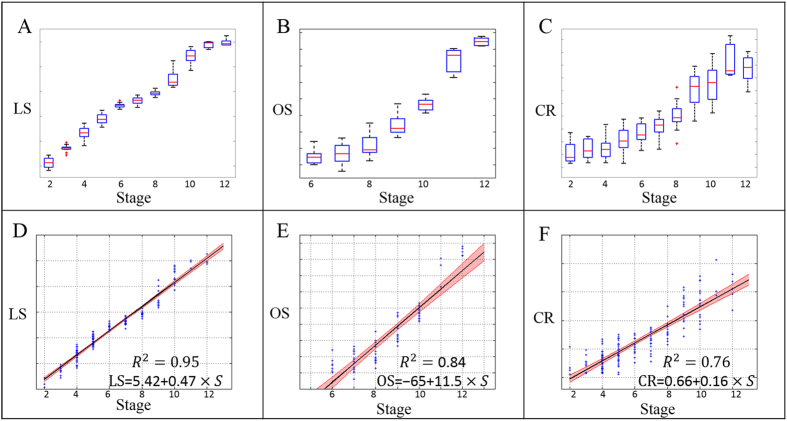
Boxplot and regression analysis. Boxplot showed the medians and dispersions of samples within each stage group; regression analysis modeled the relationship between stages and feature. (**A**) Boxplot of logarithm of egg chamber size (LS) of stage 2 to 12. (**B**) Boxplot of oocyte size (OS) of stage 6 to 12. (**C**) Boxplot of egg chamber ratio (CR) of stage 2 to 12. (**D**) Linear regression of stage (2-12) versus LS, with a coefficient of determination as R^2^ = 0.95. Shaded region represents 95% confidence interval for predicated average LS. (**E**) Linear regression of stage (6–12) versus LS with R^2^ = 0.836. Shaded region represents 95% confidence interval for predicated average OS. (**F**) Linear regression of stage (2–12) versus LS with R^2^ = 0.76. Shaded region represents 95% confidence interval for predicated average CR.

To explore the relationship between egg chamber morphological features and stages, we first used boxplot to graphically depict groups of numerical data through their quartiles. In biology, exponential growth occurs often. Instead of visualizing the size of the egg chamber, we presented the boxplot of logarithm of egg chamber size (LS) (Fig. 5A) for stages 2 to 12. This figure showed that the logarithm of the egg chamber size increased monotonically with stage. To further assess the relationship, linear regression analysis revealed a strong linear relation between the stage and the logarithm of egg chamber size (Fig. 5D) with the coefficient of determination *R*^*2*^ = *0.95*. This strong linear relationship indicates egg chamber size has an exponential growth pattern with a constant growth rate through the developmental process. The shaded area in Fig. 5D showed the 95% confidence interval of the predicated average logarithm size of the egg chambers. The two boundary lines of the shaded area were close to the fitted regression line, another indication of the strong linear relationship.

Another important egg chamber morphological feature is oocyte size (OS) (measured in terms of percentage of egg chamber, for stage 6–12), which showed a similar monotonically increasing pattern (Fig. 5B). The regression model (Fig. 5E) gave *R*^*2*^ = *0.84*. However, we observed that the OS in late stages (stage 11 and stage 12) increased relatively faster than those from previous stages, the timing of change in rate increase was consistent with the start of the nurse cell dumping process. Nurse cell dumping is a swift biological process to feed the oocyte by transferring materials into it within a short period of time^1^,^50^.

The egg chamber ratio (CR) also presented a linear pattern with respect to increasing stage (Fig. 5C, F). This quantitative evidence showed that the global shape of the egg chamber elongates from a sphere to an ellipsoid with maximal respective ratio around 3.

### Stage 8 and stage 9 follicle cell distributions

**Figure 6:**
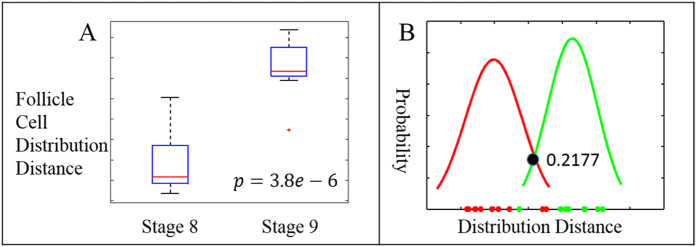
Analysis of follicle cell distribution distance between stage 8 and 9. (**A**) Boxplot of follicle cell distribution distance of stage 8 and 9. Two-sample *t*–test gives significant value *p* < 0.000004. (**B**) Fitted Gaussian curves of follicle cell distribution distance for stage 8 (red) and 9 (green). The data pointed on the horizontal axis depicted all follicle cell distribution distances (∆-distance), the range of the ∆-distance is from 0.0184 to 0.4192. The Gaussian curves were fitted by using Gaussian distribution with computed sample mean and sample standard deviation. The cutoff to classify stage 8 from stage 9 was computed as the intercept of two fitted curves. The corresponding follicle cell distribution distance of this intercept point is 0.2177.

At transitional stages 8/9, anterior follicle cells start to stretch and migrate away from each other at a wider distance^1^,^51^. The onset of anterior follicle cell stretching is closely linked to the border cell migration happening in the same period of time, which is a well-established model system to study cancer metastasis^29^,^30^. However, no one has yet clearly defined the starting point of stage 9. Here, we present a method to quantify the uniformity of the follicle cells and approach a clear definition of stage 9 onset. A two-sample *t-*test was used to assess the statistical significance of the difference between two group means. To evaluate the follicle cell distribution of stage 8 (n = 11) and stage 9 (n = 9), the distance between individual distributions of follicle cells to the uniform distribution were used in the test. The boxplot (Fig. 6A) showed a clear increase in these distance measurements from stage 8 to stage 9. The *t-*test confirmed this result with *p* = 3.79*10^−6^. This *p*-value indicated the difference between those two stages is statistically significant. The cutoff boundary of the two groups was given by ∆-distance = 0.2177, which was the intercept of the fitted Gaussian curves as shown in Fig. 6B. This means we can classify an individual egg chamber as stage 9 when the ∆-distance of its follicle cells is greater than 0.2177; otherwise, it is classified as stage 8.

### Stage classification (ordinal regression)

An appropriate machine learning algorithm for a given application is chosen depending on many factors. Two crucial factors are the given structure of data and the loss function of interest, as they simultaneously characterize the need for developing specific algorithms. In our case, we were interested in classifying the stages of egg chambers based on quantitative morphological features while the stages exhibit a natural growth order. Thus, the problem setting fits smoothly in the field of ordinal regression^52^. To apply ordinal regression analysis, we used *s* to represent the stage. The equation in Supplemental Table 1 was used to calculate the value 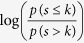, where 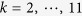, and 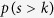
indicated the probability that a certain sample came from a stage later than *k*. The boundary cutoffs between stages were computed from those formulas, and egg chamber size cutoffs were shown in Fig. 1. Some of the values were not available, such as egg chamber size cutoff between stages 11 and 12, which was attributed to the indistinguishability in the sizes of stage 11 and 12. This was supported by the small differences in the intercepts of the last two egg chamber stages in Supplemental Table 1 as compared to other intercept differences between any other egg chamber stages in the first row of Supplemental Table 1.

## Experimental Applications

### Stage identification of the appearance of Broad expression

**Figure 7:**
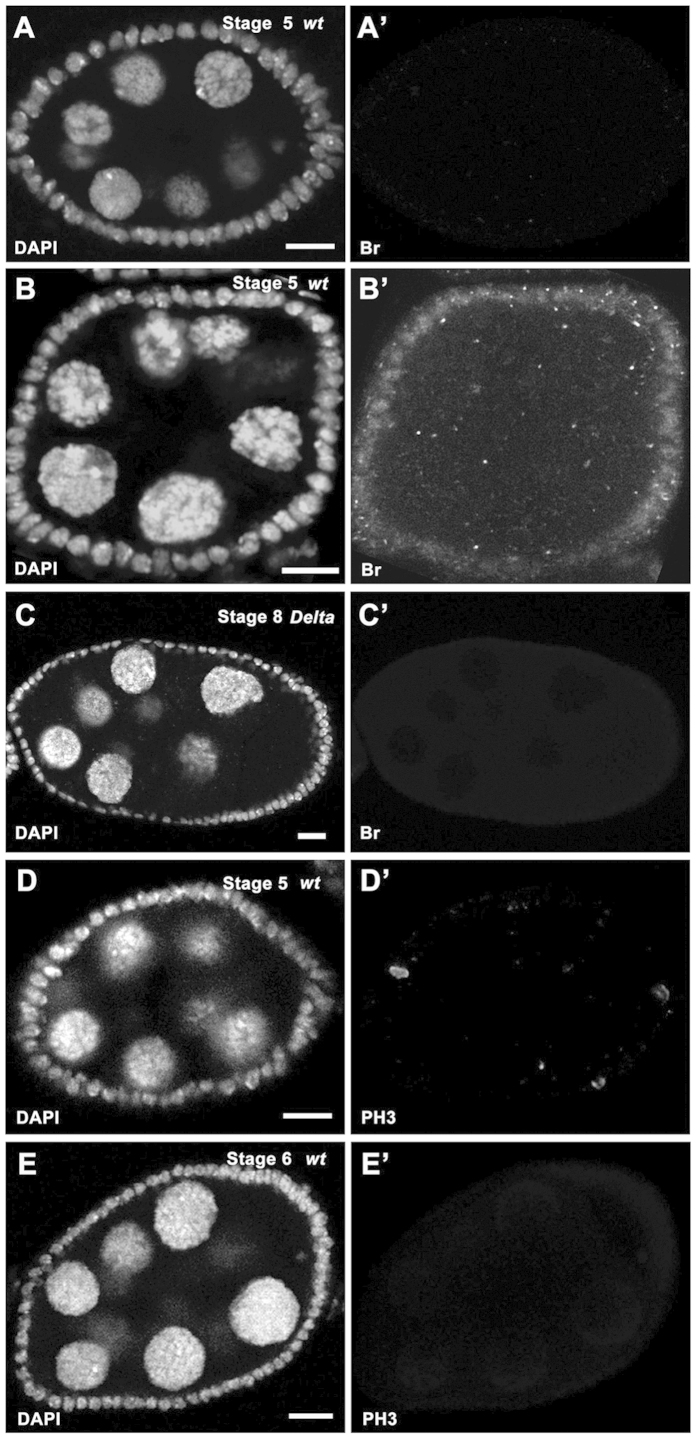
Experimental applications of stage identification. In all panels, DAPI staining (white in **A–E**) marks cell nuclei. (**A-A’**) Br expression (white in **A’**) was absent in a stage-5 wildtype egg chamber (egg chamber size: 2273.6 μm^2^). (**B-B’**) Br expression (white in **B**’) was detected in a stage-5 wildtype egg chamber (egg chamber size: 2175.4 μm^2^). (**C-C’**) Follicle cells covering the *Dl*^*rev10*^ germline clone in a stage-8 egg chamber (egg chamber size: 7908 μm^2^) did not show detectable Br expression (white in **C’**). (**D-D’**) Mitotic marker PH3 staining (white in **D’**) was detected in follicle cells of a stage-5 egg chamber (egg chamber size: 2999.4 μm^2^). (**E-E’**) Mitotic marker PH3 staining (white in **E’**) was absent in follicle cells of a stage-6 egg chamber (egg chamber size: 4606.1 μm^2^). Anterior is to the left. Bars, 10 μm.

During egg chamber development, a germline ligand, Delta, induces somatic Notch signaling from stage 5^2^, and Notch signaling directly regulates the expression of its downstream target, *broad* (*br*), in follicle cells. During the process, the N^icd^-Su(H)-Mam trimeric complex directly binds to the Su(H) binding site located at the *br* enhancer, *brE,* region^5^. Br protein level is highly upregulated at stage 6, yet it has been reported that Br could be detected at low dosage as early as stage 5 based on egg chamber morphology^5^. To confirm these findings, we examined some validated stage-5 egg chamber images by our feature extraction algorithm, and found that a few egg chambers (20%, n = 10) did not show Br expression (Fig. 7A), while a majority (80%, n = 10) had early Br presence (Fig. 7B), confirming the existence of Br from stage 5, and suggesting Br is very sensitive to Notch signaling.

### Confirmation of egg chambers with germline *Delta* mutation entering midoogenesis

The expression pattern of Br could be detected as early as stage 5, and later on, and is usable as one marker of midoogenesis (post-stage 5). However, in germline *Delta* clones, Br expression was suppressed^5^. That the expression of Br was no longer a reliable marker to label post-stage 5 egg chambers, posed a problem, and this problem applies to other cell-stage markers as well. Our toolbox provides an alternative approach to determine accurate stages. For instance, in Fig. 7C, while there was no detectable Br in the egg chamber within the germline *Delta* clones, our data extracted from DAPI images based on the computational model successfully predicted that this egg chamber was actually in stage 8 (Fig. 7C), which belongs to midoogenesis^53^, consistent with the general germline cell morphology of the egg chamber.

### Clarification of the cease of mitosis in stage-6 egg chambers

During oogenesis, the follicle cells sequentially undergo three distinctive cell cycle programs: the mitotic cycle (early oogenesis), endocycle (midoogenesis), and gene amplification (late oogenesis)^53^. Some researchers considered that the mitotic cycle includes stages 1–6, endocycle stages 7–10A, and gene amplification stages 10B-13^3^,^4^,^10^,^34^,^54^, while others believed the mitotic cycle only includes stages 1–5^1^,^2^,^38^. We applied this computational method to egg chamber images that were stained with the mitotic marker, PH3, to detect mitosis in follicle cells. We found that stage-5 egg chambers (100%, n = 17) had PH3 staining (Fig. 7D), suggesting follicle cells still underwent the mitotic cycle at that stage. Consistently, stage-6 egg chambers (100%, n = 15) showed the absence of PH3 staining (Fig. 7E). It seems clear that mitosis ceases from stage 6, and therefore endocycle should be considered to start from stage 6 as well. Notch signaling is known to appear from stage 5, and induce the mitotic cycle/endocycle (M/E) switch. However, its activation receives gradual response. Some downstream genes like *br* respond early^5^, others respond later, including Cut^3^. Egg chambers take time to coordinate signaling output to induce the M/E switch, and the differentiation of the follicle cells becomes apparent from stage 7^2^,^6^. This complex coordination process might start the M/E switch in the follicle cells at stage 5, successfully inhibit mitosis at stage 6, and fully enter in endocycle and induce differentiation at stage 7. Therefore, we propose the M/E switch occurs at stages 6/7.

## Discussion

The *Drosophila* egg chamber is a widely used model system in life science. Biologists determine the specific stages of the egg chamber mainly based on egg chamber morphology and some stage-specific markers. However, visual stage determination can be a very tedious and laborious task. Moreover, it is difficult to reproduce exactly the same measurements using manual methods, which is likely due to disparate criteria from one image to another image. Our study applies automated stage identification of the egg chambers using DAPI images in order to provide an objective and reliable method for quantifying egg chamber features and classifying stages. The method proposed here includes the integration of image processing and statistical inference. As yet, there is currently no automated method for this task, so this novel approach to stage identification offers significant merits such as objective and replicable results with minimum user interaction. Our automated method processes and counts all images using set criteria, making it immune to inter-observer and intra-observer errors.

Through linear regression analysis, egg chamber stage has shown strong correlation with all three main morphological features, logarithms of egg chamber size, oocyte size and egg chamber ratio (R^2^ = 0.95, R^2^ = 0.84 and R^2^ = 0.76, respectively). Two reasons might explain the relatively low correlation for oocyte size and egg chamber ratio. First, the DAPI image is only a cross section of the 3D structure of the egg chamber, and capture of nurse cell positions is subject to the orientation of the egg chamber around its anterior-posterior axis. Second, shearing-stress during egg chamber slide preparation may distort the egg chamber, and egg chamber ratio is sensitive to resulting distortion. These two reasons weaken the stability of egg chamber ratio compared to egg chamber size. The absolute measurements (μm^2^) of egg chamber size also bypass the limitation of various resolutions of DAPI images. The strong linear relationship between the stages and the logarithm of egg chamber size indicates an exponential growth of the egg chamber size with a constant growth rate. A close inspection of Fig. 5D reveals a decrease in growth rate from stage 6 to stage 8, thus we hypothesize there might be a growth control mechanism to slow the growth. Interestingly, there is a nutrition checkpoint at stages 8/9; if egg chambers lack adequate nutrition at this point, they then undergo apoptosis^55^. Therefore, it is likely that egg chamber growth slows from stage 6 to stage 8 in order to prepare for the nutrition check.

While our method is exciting and important to the development of standards, we admit it has limits. First, since our major goal is to propose image feature extraction methods, the DAPI images used in this study are single cross sections in the middle plane which represent the largest egg chamber size, and current code was developed on cropped images which contain one single egg chamber of interest. This limitation can be overcome by involving batch analysis of z-stack images to detect the DAPI images of cross sections with the largest area. Preprocess of egg chamber segmentation could further help us to eliminate the cropping. Second, a certain level of image quality is required for our image feature extraction process. To accommodate images with different quality levels, we provide visualization aids for results and allow user intervention when the automatically learned parameters fail to provide a proper decision. Third, in this study, we only included samples from stage 2 to stage 12. Stage-1 egg chambers are contained in the germaria, and are not separable for feature extraction. Size variations in stage-13 egg chambers are larger than the size variance in other stages. However, these problems can be easily solved by manual identification due to significantly distinguishable egg chamber morphological features of stage 1 and 13. In addition, detection of blob-like chromosomes in polytene nuclei and centripetal cell migration currently requires the cross section of the DAPI image which clearly showed stage-specific features. We also admit that skilled scientists can easily determine the stages, and routinely assign stages when they examine phenotypic or gene expression. However, scientists might assign stages differently due to human bias, and our method provides an alternative approach to determine the egg chamber stages in a repeatable and standardized way, in addition to routine descriptive guidance. Our current method is the beginning step, and we hope to involve more image/shape analysis experts and statisticians into basic biology research field. Our long-term goal is to involve the teaching process with the computer, which can literally learn from the involvement and improvement of our algorithm, and be able to adapt from manual feedback. It is similar to the deep learning concept.

The image analysis technique can be applied to other biological problems. Quantitative analysis of this type would provide objective judgment of a variety of biological phenomena. When combined with advanced machine learning and data mining techniques, quantitative measurements may further encourage discovery of complex relationships within biological events. More importantly, our image analysis toolbox provides a reliable way to standardize identification of egg chamber stages, which can integrate results across different laboratories and build up new findings on organized data. Considering these advantages, we believe the versatile utility of our algorithm outweighs its limitations.

## Materials and Methods

### Fly strains and genetics

The following fly strains were used: *hsFLP;;FRT82Bubi- RFP*, *FRT82BDl*^*rev10*^*/TM6B* (amorphic allele)^5^. *w*^*1118*^ was used as a wild-type control, cultured with standard Bloomington medium, and fed with yeast paste two days before dissection. For FLP/FRT clone induction^56^,^57^ and slide preparation, previously described procedures were followed^3^,^58^.

### Immunohistochemistry and image acquirement

Immunohistochemistry and image acquisition were carried out as previously described^3^. The following primary and secondary antibodies were used: mouse anti-Br-Core (25E9) (1:30; Development Studies Hybridoma Bank, USA), rabbit anti-PH3 (1:200; Millipore), Alexa Fluor secondary antibodies (1:400; Invotrogen). DAPI (1:500; Invitrogen) was applied to stain nuclei. Images were acquired with a Zeiss LSM 510 confocal microscope. The DAPI images should be single cross sections in the middle plane, and represents the largest area size. Cropping of a single egg chamber was processed in Image J.

### Image processing methods

Quantitative features were extracted from DAPI images using scripts written in MATLAB (MathWorks, MA, USA). The egg chamber area was measured as the convex hull of the cells belonging to that sample. Principle component analysis on the pixel locations within the egg chamber was used to identify the P-A axis. Egg chamber ratio was quantified by the ratio of the deviation along major and minor axes. We used this measurement instead of the absolute height and width of the egg chamber to accommodate possible distortion of the egg chamber during image preparation and processing. For late stages, oocyte size boundary was identified along the middle axis passing through the midsagittal plane image of the egg chamber by aligning with the direction of P-A axis. The follicle cells and nurse cells were separated by the combination procedure of using egg chamber boundary and connected regions in the image. The distribution of the follicle cells was measured by 12 dimensional vectors representing the quantity of follicle cells within 12 equally-spaced sectors along the 360 whole circle discretization started from the anterior end. Uniformity was measured by ∆-distance between the learned distribution and uniform distribution. A watershed algorithm was applied on the separated nurse cells to detect blob-like chromosomes during the late S phases at stage 4. Detection of possible centripetal cell migration was focused on the nurse cell/oocyte boundary region. The feature extraction algorithm has been implemented and tested on Matlab 2013b and newer. This toolbox is deposited at Github for free download (https://github.com/qx0731/Morphological-features-from-DAPI-image-for-egg-chamber-stage-identification).

### Quantitative methods

The egg chamber size was measured in μm^2^. Oocyte size was measured in relative area as a percentage of the whole egg chamber size. To explore the relationship between the egg chamber features of different stages, we examined three features: egg chamber size, oocyte size and egg chamber ratio. Boxplot and linear regression were conducted on egg chamber stage and the three features, respectively. DAPI images (n = 172) ranging from stage 2 to stage 12 were used in the experiments. Ordinal regression was applied to predict an ordinal dependent variable given one or more independent variables. In this paper, we conducted three ordinal regressions, each addressing one feature with respect to egg chamber stage. Classification boundaries were computed for each feature respectively. To evaluate the follicle cell distribution of stage 8 and stage 9, a two-samples *t*-test was used to show the significant difference between these two stages, and one follicle cell classification cutoff for stage 8 and 9 was also learned by finding the intercept of two fitted Gaussian models.

## Additional Information

**How to cite this article**: Jia, D. *et al.* Automatic stage identification of *Drosophila* egg chamber based on DAPI images. *Sci. Rep.* **6**, 18850; doi: 10.1038/srep18850 (2016).

## Supplementary Material

Supplementary Information
