# Supplementary material for: Automatic stage identification of Drosophila egg chamber based on DAPI images
Source: Sci Rep. 2016 Jan 6;6:18850. doi: 10.1038/srep18850 (PMC4702167; doi:10.1038/srep18850)

Automatic stage identification of *Drosophila* egg chamber based on DAPI images

Dongyu Jia, Qiuping Xu, Qian Xie, Washington Mio, Wu-Min Deng

Supplemental Table 1. Ordinal regression result between stages and morphological features. The equations used to calculate the Value $log\left(\frac{P(s\leq k)}{P(s>k)}\right)$ in ordinal regression with logarithm of egg chamber size (LS), oocyte size (OS) and egg chamber ratio (CR). This table also shows stage transition cutoffs for egg chamber size (μm^2), oocyte size and egg chamber ratio.

Stage	2		3		4		5		6		7		8		9		10		11	
	49.18-7.73×LS	579	53.21-7.73×LS	970	58.87-7.73×LS	2026	63.83-7.73×LS	4043	65.62-7.73×LS	4690	68.21-7.73×LS	6751	70.95-7.73×LS	9482	76.94-7.73×LS	20933	83.10-7.73×LS	50200	84.32-7.73×LS	
									3.17-0.298×OS	11.2	5.80-0.298×OS	19.5	8.47-0.298×OS	28.1	12.47-0.298×OS	41.8	18.12-0.298×OS	60.9	25.57-0.298×OS	79.2
	6.36-7.59×CR	0.91	7.97-7.59×CR	1.03	9.96-7.59×CR	1.32	11.85-7.59×CR		12.55-7.59×CR	1.64	13.90-7.59×CR	1.8	15.54-7.59×CR	2.02	17.67-7.59×CR	2.315	20.10-7.59×CR		20.87-7.59×CR	2.68

Supplemental Figure 1. Another example of follicle cell distribution. (A) Original image of an egg chamber. (B) Detected follicle cells were divided into 12 equal angle sectors. (C) Visualization of follicle cell distribution. Compared with Fig. 3H, this picture showed more dramatic jump across different sectors, which indicated that the follicle cells of the egg chamber in Fig. 3 distributed more uniformly than those of this egg chamber.

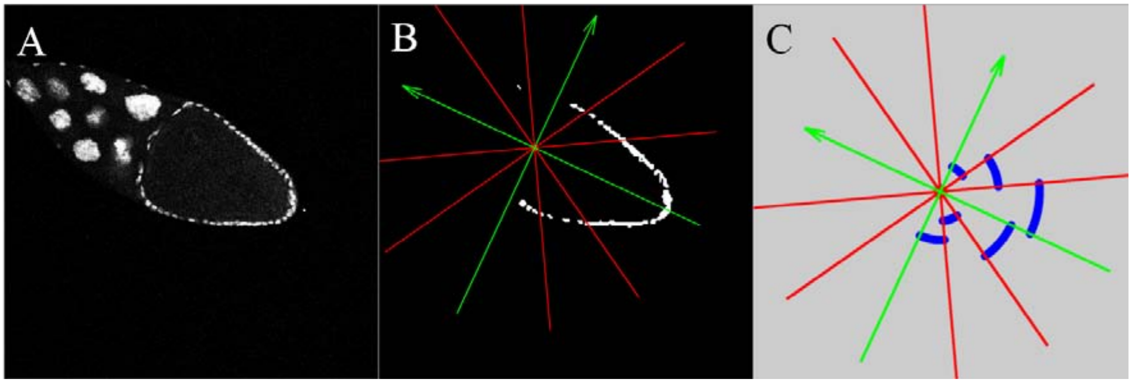

Supplemental Figure 2. Follicle cell distribution of a stage-8 egg chamber. (A) Original image of a stage-8 egg chamber. (B) Detected follicle cells were divided into 12 equal angle sectors. (C) Visualization of follicle cell distribution.

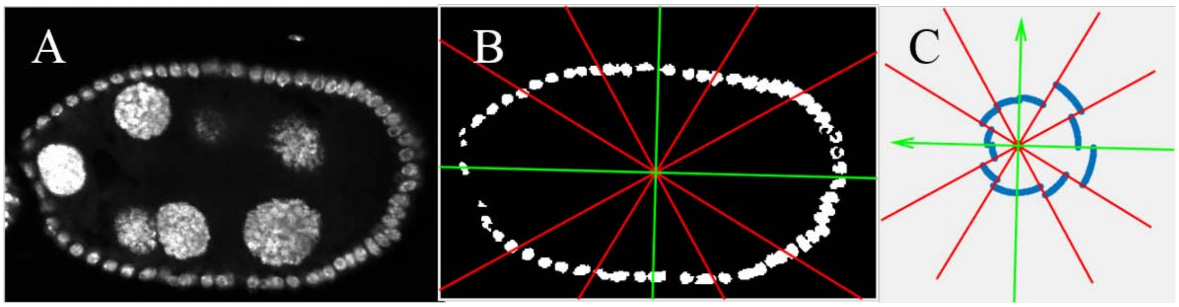

Supplemental Figure 3. Follicle cell distribution of a stage-9 egg chamber. (A) Original image of a stage-9 egg chamber. (B) Detected follicle cells were divided into 12 equal angle sectors. (C) Visualization of follicle cell distribution. Comparison between Supplemental Figure 2C with Supplemental Figure 3C showed that the more uniform distribution of follicle cells, the better circle-like visualization.

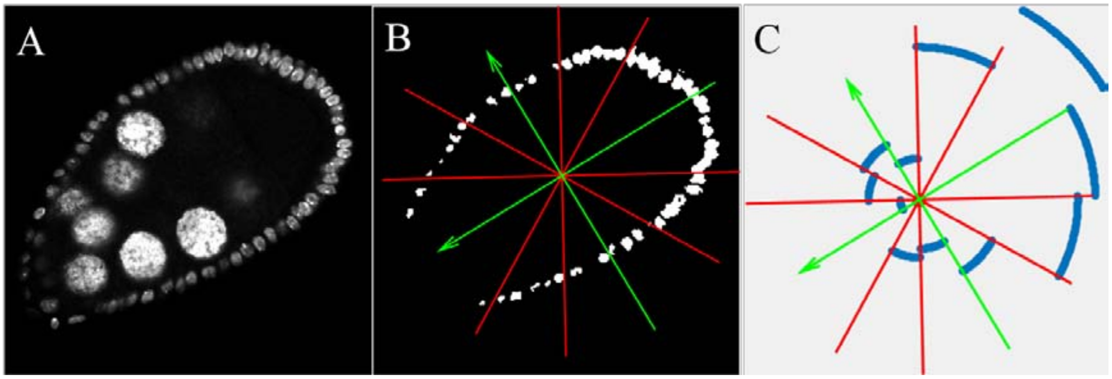

Supplement: Supplementary Information [file srep18850-s1.pdf]
